# A Case of Prostatic Carcinoma Manifesting as Cutaneous Facial Nodule

**DOI:** 10.1155/2018/5265909

**Published:** 2018-02-26

**Authors:** Israel Antonio Esquivel-Pinto, Bertha Torres-Alvarez, Ramiro Jetzabel Gómez-Villa, Juan Pablo Castanedo-Cázares

**Affiliations:** Department of Dermatology, Hospital Central Dr. Ignacio Morones Prieto, Universidad Autónoma de San Luis Potosí, San Luis, SLP, Mexico

## Abstract

Prostate cancer is the second most frequently diagnosed cancer worldwide and the fifth most common cause of cancer deaths among men. Cutaneous metastasis is an uncommon phenomenon in prostatic cancer, occurring in 0.06–0.3% of cases.* Case Presentation.* A 56-year-old man presented to our outpatient clinic with a one-month history of a 1.5 cm in diameter, solitary, asymptomatic, purple nodule located on his upper right cheek. After biopsy, prostatic cancer metastasis was diagnosed.* Discussion.* A literature review revealed 59 articles documenting 71 cases of this diagnosis. The review recorded epidemiological data, including age, duration, morphology, location, and outcome of patients.* Conclusions.* The skin is an uncommon site for metastasis of prostate cancer, and the review showed that its presence is associated with a poor prognosis (approximately 10 months from diagnosis).

## 1. Introduction

Prostate cancer is the second most frequently diagnosed cancer worldwide and the fifth most common cause of cancer deaths among men. In 2012, 1.1 million new cases were diagnosed globally, and more than 300,000 deaths were related to this disease [1]. Its incidence is directly related to the aging population and their socioeconomic development. In developing countries like Mexico, the annual incidence is approximately 6–13 cases per 100,000 inhabitants [2]. Prostate carcinoma may present skin metastases in 0.06–0.3% of cases [3–5]. A cutaneous lesion manifesting a tumor without urologic symptoms is a rare event.

## 2. Case Report

**Figure 1:**
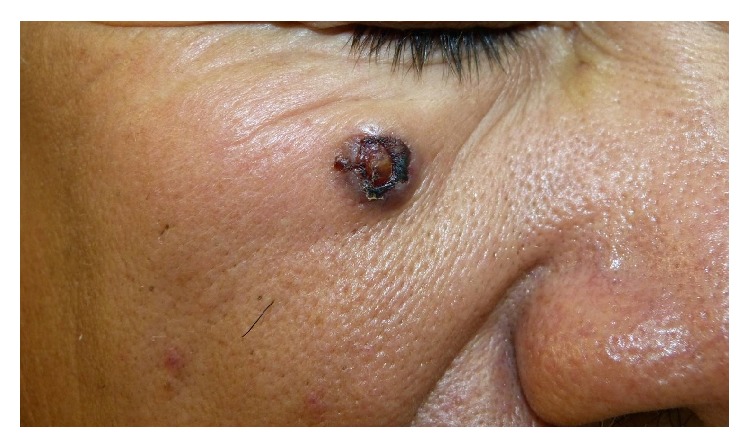
Clinical image of lesion. A purple nodule, located on the right cheek, with a diameter of 1.5 cm and an ulcerated center and surrounded by hematic crust having slightly raised edges and a firm consistency.

A 56-year-old man, who was a farmer without relevant medical history, presented to our outpatient clinic with a one-month history of a 1.5 cm in diameter, solitary, asymptomatic, purple nodule located on his upper right cheek. It had a firm consistency, an ulceration in its center, and slightly raised edges surrounded by hematic crusts (Figure 1). He did not refer malaise or unusual osteomuscular pain but alleged an unintentional loss of weight of approximately 8 kg in the last 10 months without seeking medical attention. Upon further interrogation, the patient mentioned that he had experienced transitory urinary symptoms, including dysuria, frequency, urgency, and urinary retention, one year earlier, but they had resolved spontaneously. Examination of his oral cavity, neck, regional lymph nodes, and the rest of his skin was unremarkable. During urologic exploration, a manual examination of the prostate determined its size to be 40 × 55 mm, with an indurated mass inside without tenderness.

Based on the clinical history and physical examination, diagnoses considered were pyogenic granuloma, amelanotic melanoma, clear cell acanthoma, and basal cell carcinoma. An excisional biopsy was performed. During the 24 hours after surgery, there was continuous bleeding at the biopsy site, so conservative treatment by compression of the site was indicated. After 48 hours, mild hemorrhaging persisted, so the stitches were removed and the wound was explored for the origin of the blood loss. No underlying vessel was found, so vertical mattress sutures were applied to stop the bleeding. We requested blood tests, which evidenced normal coagulation parameters but a low platelet count of 67,000/mm^3^ and an alkaline phosphatase level of 721 UI/L were found.

**Figure 2:**
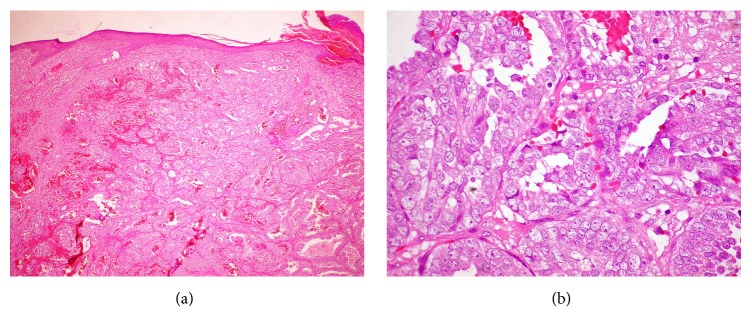
(a) The skin biopsy showed abundant hematic crust and ulcerated areas in the epidermis. Ectatic abundant vessels were observed at the entire thickness of the papillar and reticular dermis. (b) Cells with broad, abundant, clear cytoplasm in ductal arrangement were observed. This aspect was suggestive of skin metastases of glandular neoplasia, including prostate or thyroid (hematoxylin-eosin stain; original magnifications: 40x (a); 400x (a, b)).

**Figure 3:**
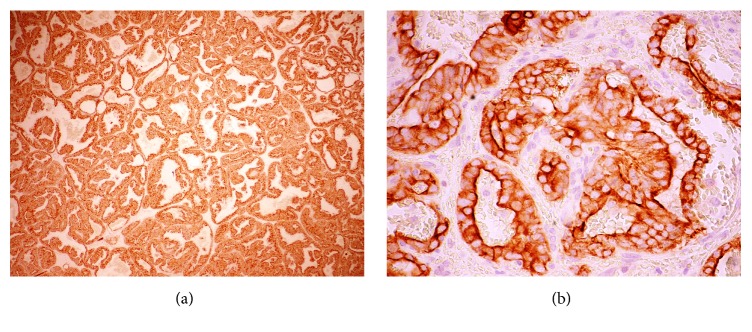
Dermal infiltrate positive for prostate specific antigen (PSA). This marker is usually positive in prostatic carcinoma (avidin-biotin-peroxidase complex staining. Original magnifications: 100x (a), and 400x (b)).

The biopsy of the skin nodule showed that the papillary and reticular dermis were infiltrated by abundant cells having broad, clear cytoplasm, a ductal arrangement, and ectatic vessels (Figures 2(a) and 2(b)). The aspect was suggestive of metastases from glandular neoplasia, probably thyroid or prostate. The thyroid function profile was normal, but the prostate specific antigen (PSA) was 1124 ng/ml (normal: 4 ng/ml for age group). Immunohistochemistry examination found neoplastic cells positive for PSA (Figures 3(a) and 3(b)), Ki-67, and CD10 in a diffuse and >10% expression, while thyroid transcription, calponin, and thrombomodulin were negative.

Computer tomography (CT) was performed to assess the extent of the neoplasia. Sections of the head showed no further evidence of metastatic lesions. However, abdominal and pelvic segments evidenced a prostate gland size of 40 × 55 mm, in addition to osteolytic lesions in the lumbar vertebral bodies. Therefore, the diagnosis of metastatic prostate ductal adenocarcinoma to the skin was established with a stage of T4, N1, and M1c, which corresponded to a disseminated cancer with poor prognosis at 6 months. The patient was referred to another institution for treatment, where he was treated with hormonal therapy and died after 8 months due to disseminated disease.

## 3. Discussion

Varied presentations of cutaneous metastases resulting from internal malignancies have been reported and described as diagnostic challenges for clinicians [2]. A review of the literature that searched the PubMed database for “skin cutaneous metastases OR or AND prostatic cancer metastasis prostate cancer” retrieved 59 articles documenting 71 cases of this diagnosis. The mean age was 67 years, with a range of 50–81 years. In 63% of cases, the diagnosis was already known, but it was not specified in 21%. In approximately only 15% of cases, skin lesions helped to establish the initial diagnosis of prostatic cancer [3–60].

The sites of metastasis included the pelvic region in 20 cases, the thorax in 10 cases, the face and neck in 8 cases, the scalp in 8 cases, the limbs in 6 cases, and unspecified sites in 9 cases. The clinical appearances of these cutaneous metastases of prostate cancer were extremely variable, as 31 presented as nodules, 8 as infiltrated plaques, 5 as Sister Mary Joseph's nodules (a palpable nodule bulging into the umbilicus), 3 as sclerodermiform lesions, 3 as vascular proliferations, 3 as abscesses, 2 as cystic, 2 as zosteriform, and 14 as undescribed. Most cases (65%) presented 2 or more cutaneous lesions at the time of diagnosis [3–61].

The histopathologic features of the cutaneous metastatic prostate carcinomas usually were similar to the primary tumor and helped establish the diagnosis. Some features included sheets of undifferentiated cells diffusely infiltrating the dermis, often with a conspicuous Grenz zone; gland-like structures if the primary tumor was of the adenocarcinoma type; and subtle, nuclear atypia characterized by hyperchromasia and prominent eosinophilic nucleoli, although adnexal structures may likewise be infiltrated by malignant cells [3, 9]. Immunohistochemistry was useful to confirm the etiology of the primary tumor in equivocal cases. PSA is a protein produced by cells of the prostate gland and was the most commonly employed stain, as nearly 97% of metastatic prostate carcinomas are positive for this protein [62]. The expression of human Ki-67 protein and CD10 are helpful to the study of suspected metastatic lesions because they are strictly associated with cell proliferation, aggressive migration, and advanced disease [63, 64].

We consider our case important, as the cutaneous expression of a prostatic carcinoma is an infrequent phenomenon, occurring in only 0.06–0.3% of cases. The patient we describe presented an isolated nodule on his face. This was an uncommon morphology and an uncommon site for this metastasis, as only 11% of cases are situated in this area, and the most implicated skin site, accounting for up to 35% of these events, is close to the inguinal region [2]. In addition, this lesion on the cutaneous area of the right cheek was accompanied by no current urologic symptoms, but only by a low platelet count and high levels of alkaline phosphatase. These abnormal parameters were promptly explained by the bone invasion documented by CT, as platelet-count alterations and blood release of alkaline phosphatase may be manifestations of osseous and/or liver metastatic disease [65].

Skin metastasis is a complex process whereby tumor cells acquire the necessary biological properties enabling them to invade it from the primary lesion. These cells spread by lymphatics and/or the bloodstream, survive in the circulation, and extravasate into the dermis to proliferate at the new site [2]. Although the dispersion mechanism in our patient, from genital area to face, is uncertain, it could be explained by its access through the Batson's plexus, a venous system that connects from the vertebral to supradiaphragmatic, cervical, and head veins [66]. Chymotrypsins, including the human tissue kallikrein 7 (hK7), a secreted serine protease that catalyzes the degradation of intercellular adhesive structures in the cornified layer of the skin, may be involved in the cutaneous manifestation of this neoplasm [66, 67]. Other selectin and receptors may be implied, including the cytosolic R1881 and androgen receptors, although further studies are needed to discern the cutaneous metastatic prostate process [68, 69].

Therapeutic options reported for similar cases of prostatic carcinoma with cutaneous metastasis are primarily palliative and include tumor excision, radiation, intralesional chemotherapy (i.e., leuprolide), and treatment of the primary neoplasia (i.e., chemotherapy, hormonal, or surgical) [3–60]. Such therapies must be carefully selected on a case-by-case basis, but it is notable that treatment of the primary malignancy results in partial improvement of cutaneous metastases in 65% of cases in a period of 4–8 weeks after treatment is begun [3–60].

Although cutaneous metastasis is an uncommon presentation of prostate cancer, it remains an important diagnostic consideration in patients with unrecognized, advanced disease. It should always be considered in cases of rapidly growing cutaneous nodules, especially if they are associated with past or current urologic symptoms and particularly if they are accompanied by persistent bleeding such as that which occurred in our patient. Thus, the suspicions of early interventions by dermatologists, assisted by further histologic studies of cutaneous lesions, play an important role in diagnosis and establishing early treatment, as the mean survival time for the reviewed cases of prostate cancer was approximately 10 months after the onset of skin involvement [3–60].
